# Combined Photodynamic Therapy and Chemotherapy Using Local Intra-Arterial Intratumoral Administration of Chlorin e6 and Cisplatin: First Clinical Observations

**DOI:** 10.3390/ijms26178640

**Published:** 2025-09-05

**Authors:** Kanamat Efendiev, Artem Shiryaev, Aidai Rakhmanova, Tatiana Pisareva, Alena Mamedova, Svetlana Samoylova, Igor Reshetov, Alexey Skobeltsin, Anna Krivetskaya, Anastasia Ryabova, Vladimir Makarov, Victor Loschenov

**Affiliations:** 1 Prokhorov General Physics Institute of the Russian Academy of Sciences, 119991 Moscow, Russia; 2 Department of Laser Micro-, Nano-, and Biotechnology, Institute of Engineering Physics for Biomedicine, National Research Nuclear University “MEPhI”, 115409 Moscow, Russia; 3 Department of Oncology, Radiotherapy and Reconstructive Surgery, Levshin Institute of Cluster Oncology, Sechenov First Moscow State Medical University, 119435 Moscow, Russia; 4 Laboratory of Neurobiology and Tissue Engineering, Brain Science Institute, Research Center of Neurology, 125367 Moscow, Russia

**Keywords:** photodynamic therapy, chemotherapy, photosensitizer, chlorin e6, chemotherapeutic agent, cisplatin, intra-arterial administration, fluorescence diagnostics, head and neck squamous cell carcinoma, extracellular matrix, targeted therapy

## Abstract

Despite advances in cancer treatment, head and neck squamous cell carcinoma (HNSCC) remains a serious clinical problem due to tumor aggressiveness, tumor resistance to therapy, and treatment toxicity. The combination of photodynamic therapy (PDT) with chemotherapy is a promising approach to improve efficacy while reducing side effects. For the first time, the possibility and antitumor effect of the combined use of PDT and chemotherapy with intra-arterial administration of chlorin e6 (Ce6) and cisplatin in patients with HNSCC were assessed. Two patients with locally advanced HNSCC received intra-arterial administration of Ce6 (at a dose of 0.5 mg/kg) and cisplatin (at a dose of 50 mg/m^2^) via a catheter into the tumor-feeding artery followed by laser irradiation. Ce6 distribution, tumor response, and treatment efficacy were assessed by fluorescence diagnostics, confocal microscopy, and histopathological analysis. Intra-arterial administration of the photosensitizer (PS) and chemotherapeutic agent ensured high selectivity of their tumor accumulation. Fluorescence diagnostics showed rapid and selective Ce6 accumulation in the tumor and PS photobleaching after PDT. For a patient with three PDT sessions, there is a significant acceleration of the Ce6 spread from the tumor-feeding artery throughout the tumor bed with each therapy session. This is a good sign of a tumor stroma density decrease. The combined use of PDT and chemotherapy with intra-arterial administration of Ce6 and cisplatin is safe and feasible, with preliminary evidence of local cytotoxicity treatment for HNSCC, allowing targeted drug delivery to the tumor. This is the first report of the combined use of PDT and chemotherapy with selective intra-arterial administration of a PS and a chemotherapeutic drug for the treatment of cancer.

## 1. Introduction

Cancer is one of the leading causes of death in the world. According to the World Health Organization (WHO), in 2022, there were about 20 million new cases of cancer and more than 9.7 million deaths from cancer worldwide, which corresponds to a lifetime risk of developing cancer of about 1 in 5 and a risk of dying from it of 1 in 9 for men and 1 in 12 for women [1]. Among all sites, head and neck squamous cell carcinoma (HNSCC) accounts for approximately 890,000 new cases (4.5% of all cancer diagnoses) and 450,000 deaths (4.6% of all cancer deaths) annually [2]. Lip and oral cavity cancer ranks 16th in frequency among all malignancies, with 389,846 new cases and approximately 180,000 deaths in 2022 [3,4]. The 5-year survival rate for localized HNSCC is 86.6%, compared to 69.1% for locally advanced HNSCC and 39.3% for metastatic HNSCC [2].

During the carcinogenesis of HNSCC, the composition and density of the extracellular matrix (ECM) undergo significant alterations. In the early stages of HNSCC progression, newly transformed epithelial cells trigger a wound-like response, leading to stromal fibroblast activation and the accumulation of matrix proteins, ultimately resulting in ECM compaction [5]. The capacity of tumor cells and tumor microenvironment (TME) cells to synthesize components of the ECM critically influences tumor progression [6]. The dense ECM creates a “protective shield” that significantly impairs antitumor agent efficacy [7]. Furthermore, by compressing blood vessels, the ECM reduces vascular delivery of these therapeutics to tumor cells. This vascular compression induces local hypoxia, subsequently activating anti-apoptotic pathways and promoting neo-angiogenesis [8].

Despite significant progress in developing early diagnostic and treatment methods, many types of cancer are characterized by high aggressiveness, a tendency to metastasize, and resistance to standard treatments. Currently, some of the most common methods of cancer treatment are surgical removal of the tumor, radiation therapy and chemotherapy, a combination of chemotherapy with radiation therapy (chemoradiation therapy), or a combination of chemoradiation therapy before or after surgery. Surgical removal of the tumor is often limited by the size of the tumor and its location, since radical resection can lead to significant functional impairment, a decrease in the patient’s quality of life, and a pronounced cosmetic defect, which is especially important in the treatment of head and neck tumors. Surgical resection in the head and neck area is often accompanied by speech and swallowing dysfunction, cosmetic defects, and a decrease in the quality of patients’ life [9]. Chemotherapy, being one of the most frequently used systemic strategies for the treatment of malignant neoplasms, can have a cytotoxic effect on both primary tumor foci and metastases [10]. However, chemotherapy has several significant limitations. These include a non-specific effect on healthy tissues, pronounced side effects and the formation of resistance of tumor cells to chemotherapy agents [11]. Radiation therapy and chemoradiation therapy also play an important role in the treatment of HNSCC [12]. However, the radiation load on the body during radiation or chemoradiation therapy remains a serious clinical problem [13], as it can be associated with the risk of later complications in patients [14]. Radiation therapy can cause severe acute and late complications: acute mucositis and dysphagia (up to 40% of patients in different studies), xerostomia in almost all patients and soft tissue fibrosis [15,16]. Both treatment methods can also cause numerous side effects in the oral cavity, paranasal sinuses, and pharynx, which adversely affects the quality of life of patients [12]. All these factors stimulate the search for new therapeutic approaches and the development of combined methods that would improve the effectiveness of cancer treatment and reduce toxic effects on normal tissues and the patient’s body.

**Figure 1:**
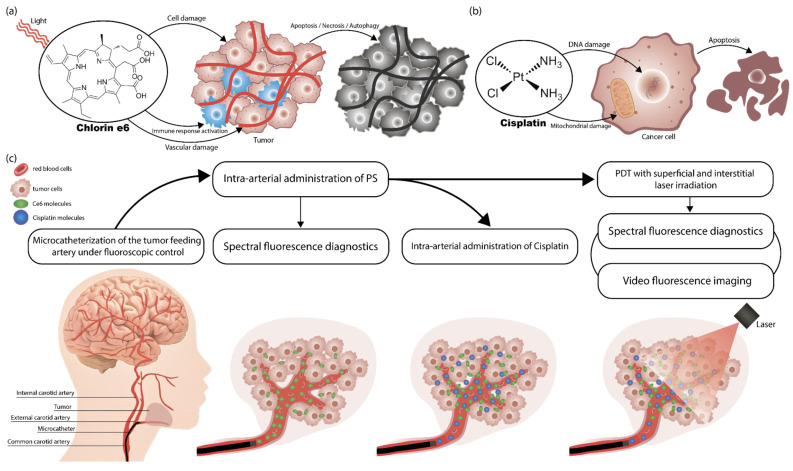
Mechanisms of antitumor action: (**a**) Ce6-mediated PDT; (**b**) cisplatin-based chemotherapy; (**c**) scheme of diagnostics and treatment with combined use of PDT and chemotherapy with selective microcatheterization of the tumor-feeding artery and intra-arterial administration of Ce6 and cisplatin. The scheme entails the following sequence of actions: firstly, microcatheterization of the tumor-feeding artery is performed under fluoroscopic guidance; then, a PS is administered intra-arterially; subsequently, intra-arterial administration of cisplatin is initiated; finally, PDT is performed during the administration of cisplatin.

In recent years, minimally invasive methods that combine photodynamic therapy (PDT) have been actively used due to their high efficacy and selective effect on tumor cells [17,18]. PDT includes two main stages. First, photosensitizer (PS) is administered to the patient’s body. The PS molecule has the property of selectively accumulating in tumor tissues. Second, the sensitized tissues are exposed to light of a specific wavelength that corresponds to one of the PS absorption peaks. Light irradiation triggers the process of generation of reactive oxygen species (ROS) [19]. ROS have high reactivity and short half-life, which limits their diffusion into normal cells [20]. ROS generated during photodynamic therapy (PDT) can destroy tumor cells directly by causing apoptosis, necrosis, and/or autophagy. They can also destroy the tumor vascular system, disrupting the oxygen supply and activating the immune response against tumor cells [21,22] (Figure 1a).

The efficacy of PDT depends on all these mechanisms of action, and the contribution of each of them is determined by the PS types and light irradiation modes used. PDT is limited by the depth of light penetration, which is usually ≤1–2 cm, and in some cases, by the low selectivity and heterogeneity of PS accumulation in the tumor. Clinical studies of early-stage head and neck tumors have shown an overall response rate of 85–100%, with up to 75% of patients achieving a complete response that persists two years after PDT treatment [23]. The significant advantages of PDT encompass local and multiple treatments and the absence of resistance formation by tumor cells.

The most commonly used chemotherapeutic drugs in cancer treatment today are platinum (II) (Pt(II))-based agents, including cisplatin (cis-diamminedichloridoplatinum (II), CDDP), carboplatin (cis-diammine-1,1-cyclobutanedicarboxylatoplatinum(II), CBDCA), and oxaliplatin (trans-R,R-cyclohexane-1,2-diamineoxalateplatinum(II), 1-OHP) [24]. Pt(II)-based agents exert their cytotoxic effect primarily through the formation of intrastrand deoxyribonucleic acid (DNA) crosslinks and, to a lesser extent, interstrand crosslinks [25]. Such DNA damage alters the double-helix structure, triggering cell cycle arrest via DNA damage response pathways and ultimately leading to tumor cell apoptosis (Figure 1b). These agents also have known secondary mechanisms of action, such as effects on mitochondrial biogenesis [26] or ROS production [27]. Chemotherapy is frequently combined with radiotherapy or surgery. However, the main limitation is chemoresistance, which contributes to disease relapse, progression and metastasis, worsening patient outcomes. Interestingly, PDT can disrupt resistance mechanisms locally, and combining PDT with chemotherapy may enhance treatment efficacy, offering new strategies to overcome resistance and improve survival rates [28].

Recent studies have established that optimized combination protocols of PDT and chemotherapy can achieve significantly improved antitumor outcomes compared to monotherapy approaches [29]. Several in vitro studies have demonstrated the enhanced cytotoxic effects of combining PDT with Pt(II)-based chemotherapy, including (1) cisplatin with Photofrin II^®^ (Axcan Pharma Inc., Mont Saint-Hilaire, Canada) in esophageal carcinoma and with Photofrin^®^ (Lederle Japan Co. Ltd., Tokyo, Japan) in murine lymphoma models [30,31]; (2) cisplatin with methylene blue for A2780 and A2780-CP cell lines [32]; and (3) carboplatin paired with either 9-hydroxypheophorbide α (9-HPbD) or Radachlorin^®^ (RADA-PHARMA Co. Ltd., Moscow, Russia) for laryngeal cancer [33,34,35], carboplatin with Verteporfin^®^ (Alcami Carolinas Corporation, Charleston, SC, USA) for ovarian cancer [36], or Photofrin^®^ (LightpharmTech, Seoul, Republic of Korea) for cervical cancer [37]. A comprehensive evaluation of Temoporfin (mTHPC)-mediated PDT combined with carboplatin, cisplatin, or oxaliplatin (1-OHP) across multiple cancer types (bladder, cervical, esophageal, lung, and oral) revealed significant tumor tissue-dependent synergistic effects [24]. Beyond Pt(II)-based agents, the combination of zinc phthalocyanine (ZnPc)–PDT with low-dose doxorubicin (DOX) (5 J/cm^2^ irradiation) demonstrated enhanced apoptotic activity in SK-MEL-3 melanoma cells, suggesting potential for reduced chemotherapeutic dosing [38]. Innovative delivery systems have further improved combination efficacy, as evidenced by smart dual-functionalized gold nanoclusters (AuNC-PpIX-DOX) showing controlled dual cytotoxic effects in HeLa cells through combined chemotherapy and photodynamic action [39]. Recent clinical findings [40] demonstrate significantly improved 12-month survival outcomes in unresectable cholangiocarcinoma patients receiving combined chemotherapy–PDT therapy compared to either treatment modality alone. According to study [41], combined treatment modalities significantly reduced mortality risk in advanced non-small cell lung cancer patients: a 50% reduction was observed in the PDT-with-chemoradiotherapy group, compared to a 53% reduction in the chemoradiotherapy-only group.

Chlorin e6 (Ce6)-derived PSs represent one of the most clinically valuable and extensively utilized classes of agents in PDT. Recent clinical progress has highlighted the effectiveness of Ce6-based PDT approaches, particularly when combined with fluorescence diagnostic techniques [42,43,44]. Several Ce6-derived PS formulations have been successfully implemented in clinical practice, including Photolon^®^ (Republican Unitary Production Enterprise “Belmedpreparaty”, Minsk, Belarus) [43], Photoran E6^®^ (DEKO Company Ltd., Moscow, Russia) [45], Photoditazine^®^ (LLC “VETA-GRAND”, Moscow, Russia) [46], Radachlorin^®^ (RADA-PHARMA Co. Ltd., Moscow, Russia) [47], Talaporfin^®^ [48], Temoporfin^®^ (Meiji Seika Pharma Co. Ltd., Tokyo, Japan) [49], and Foscan^®^ (Scotia Pharmaceuticals Ltd., Stirling, UK) [50]. These PSs demonstrate several advantageous properties, including excellent aqueous stability and minimal dark toxicity [51], making them particularly suitable for therapeutic applications. It should be noted that many PSs, including Ce6, display appreciable accumulation in organs of the reticuloendothelial system (notably liver and spleen) and other tissues of the body [42]. Such tissue distribution is determined not only by systemic elimination kinetics but also by physicochemical properties of the compound—in particular its affinity for circulating lipoproteins and lipoprotein-mediated cellular uptake—processes that may be enhanced in neoplastic tissues by upregulation of lipoprotein receptors [52]. At the same time, the short lifetime and limited diffusion distance of ROS generated during photodynamic activation remain important factors that constrain phototoxic effects spatially when light activation is local [53].

The combination of Ce6-mediated PDT with cisplatin-based chemotherapy, particularly via the novel approach of selective intra-arterial administration of both agents directly to the tumor-feeding artery, has not been clinically reported for cancer treatment. A comparison of previous clinical studies is presented in Table 1, underscoring the novelty of our approach.

In this study, we evaluated the antitumor efficacy and safety of the combination of Ce6-mediated PDT and cisplatin-based chemotherapy in the treatment of HNSCC (buccal mucosa cancer and tongue cancer) for the first time. We used an innovative therapeutic approach utilizing selective intra-arterial administration of both a PS and chemotherapeutic agent through tumor-feeding artery catheterization for the first time. The main objective of the study was to evaluate the antitumor effect of the combined use of Ce6-mediated PDT and cisplatin chemotherapy with local intra-arterial administration of a PS and chemotherapeutic agent.

## 2. Results

**Figure 2:**
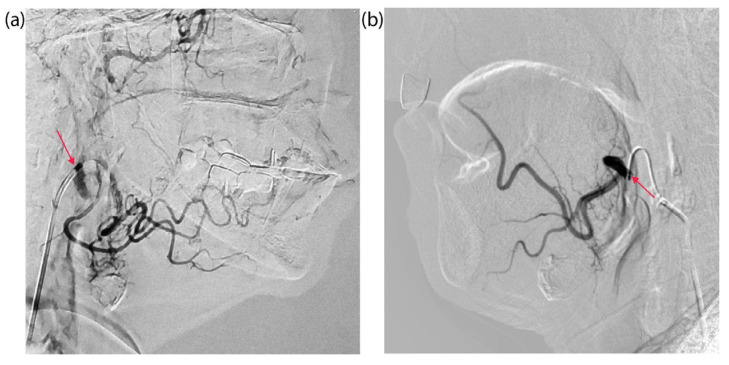
Catheterization of the arteries supplying the tumor under CT guidance: (**a**) patient KIA—installation of a microcatheter in the projection of the mouths of the facial and lingual arteries extending from the right ECA; (**b**) patient PCM—selective microcatheterization of the left facial artery through the left ECA using a microcatheter. The arrow indicates the location of the microcatheter.

Preliminary selective catheterization of the arteries supplying the tumor was performed in both patients under the control of computed tomography (CT). Figure 1c illustrates the study’s overall design. For patient KIA with tongue cancer, the microcatheter was positioned in the orifices of the facial and lingual arteries, which branch off from the right external carotid artery external carotid artery (ECA) (Figure 2a). For patient PCM with a buccal mucosa tumor, the microcatheter was positioned in the left ECA to access the left facial artery (Figure 2b).

**Figure 3:**
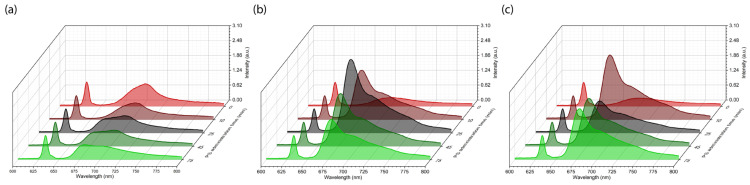
Spectra registered during spectral-fluorescence diagnostics of the patient KIA: (**a**) normal mucosa; (**b**) tumor center; (**c**) tumor border. These spectra include components of diffusely scattered excitation laser radiation ranging from 625 to 645 nm and tissue fluorescence ranging from 650 to 800 nm.

After fixation of the microcatheter, before the start of PS administration, spectral-fluorescence diagnostics was performed to register tissue autofluorescence in the range of 600–800 nm at the excitation wavelength λexc = 632.8 nm. Three anatomical zones were identified for diagnosis: tumor center, tumor border, and normal/intact oral mucosa. Spectral-fluorescence diagnostics was performed in each zone throughout the period before and after PS administration to detect maximum Ce6 accumulation. In Figure 3, the registered spectra of the patient KIA are shown from the second session of combined treatment with PDT and chemotherapy.

**Figure 4:**
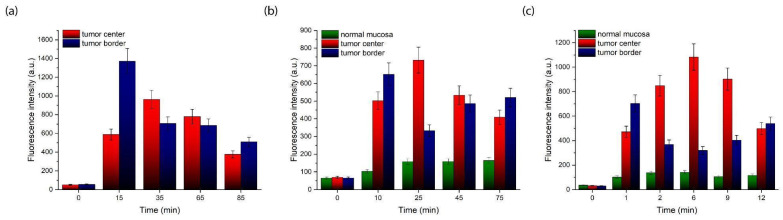
Dynamics of Ce6 accumulation in normal mucosa, tumor central, and tumor border zones of patient KIA after intra-arterial PS administration before PDT: (**a**) first session of combined treatment; (**b**) second session of combined treatment (1 month after the first session); (**c**) third session of combined treatment (2 months after the first session).

After starting intra-arterial PS administration, characteristic Ce6 fluorescence with a maximum in the range of 650–690 nm was recorded in normal oral mucosa (Figure 3a), the central tumor zone (Figure 3b), and the tumor border (Figure 3c). Because patient KIA had a tumor in the mobile part of the tongue and radical resection was initially planned, which could result in loss of tongue function, three sessions of combined neoadjuvant treatment using PDT and chemotherapy were performed with monthly intervals to minimize tumor volume for organ-preserving surgery. Figure 4 shows the distribution of integrated Ce6 fluorescence intensity in the 650–690 nm range at different stages of combined treatment for patient KIA.

In patient KIA, we observed a high selectivity of Ce6 accumulation and different Ce6 accumulation dynamics in tumor tissues at different stages of treatment (Figure 4). During the first session of combined treatment, maximum Ce6 accumulation in the central tumor zone was recorded 35 min after intra-arterial PS administration began, while maximum accumulation at the tumor border was recorded 15 min after administration began (Figure 4a). During the second session, the maximum Ce6 accumulation in the tumor’s central zone occurred at 25 min, while at the tumor border, it occurred at 10 min (Figure 4b). During the third session, the maximum Ce6 accumulation in the central tumor zone occurred at 6 min, and at the tumor border, it occurred at 1 min (Figure 4c). In all cases, maximum fluorescence was achieved faster at the tumor border than in the central zone. Furthermore, during the second and third sessions, an increase in Ce6 fluorescence intensity was observed at the tumor border after the central zone reached its accumulation peak. This may be due to the redistribution of PS (Ce6 inflow and outflow) between the central and border zones of the tumor due to blood flow and vascularization characteristics.

**Figure 5:**
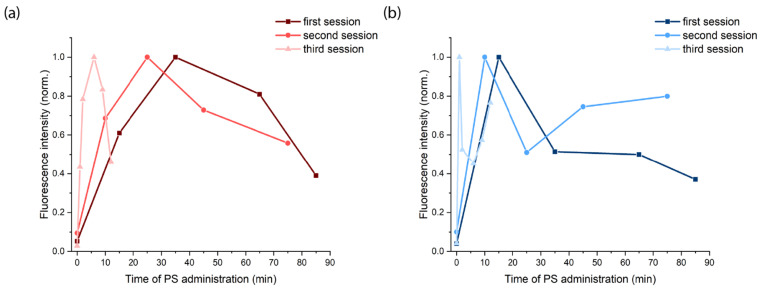
Ce6 fluorescence intensity in a tumor after intra-arterial administration: (**a**) tumor center; (**b**) tumor border. Fluorescence intensity is normalized to the maximum value.

It should be noted that the greatest contrast of Ce6 accumulation is recorded at the beginning of the PS administration. Then, the Ce6 concentrations in the central and border zones of the tumor equalize, as shown in Figure 4. Additionally, during the treatment process, a reduction in the time to reach the maximum of Ce6 fluorescence intensity is observed in both the central (Figure 5a) and border (Figure 5b) zones of the tumor, which may be associated with a decrease in the density of the ECM. We have demonstrated earlier that Ce6-mediated PDT predominantly acts through a vascular mechanism [57]. The damage to blood vessels directly during PDT should lead to a long-term retention of cisplatin in the tumor area [58], which may allow for increased diffusion of the chemotherapeutic agent from the blood vessels into the tumor parenchyma. Therefore, intra-arterial administration of cisplatin was performed directly during the PDT process (Figure 1c). Here, the Ce6 distribution time throughout the tumor volume after intra-arterial administration indicates normalization of the tissue permeability each month after PDT sessions.

**Figure 6:**
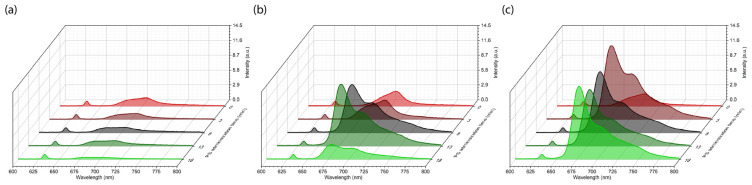
Spectra registered during spectral-fluorescence diagnostics of the patient PCM: (**a**) normal mucosa; (**b**) tumor center; (**c**) tumor border. These spectra include components of diffusely scattered excitation laser radiation ranging from 625 to 645 nm and tissue fluorescence ranging from 650 to 800 nm.

Patient PCM underwent one session of combined treatment. Figure 6 shows the results of spectral-fluorescence diagnostics during the intra-arterial administration of Ce6.

Spectral-fluorescence diagnostics were performed on the patient PCM at the following times: before PS administration (0 min) and 3, 8, 13, and 18 min after the start of PS administration. After the start of PS administration, intense Ce6 fluorescence in the range of 650–690 nm was detected in the central and border zones of the tumor (Figure 6b,c). In normal mucosa, accumulation of Ce6 was not observed (Figure 6a). The maximum Ce6 accumulation in the tumor central zone occurred at the 13th min (Figure 6b). In the tumor border zone, maximum Ce6 accumulation occurred at the 8th min (Figure 6c). After the 13th min, Ce6 fluorescence intensity increased (Figure 6c), possibly due to Ce6 inflow and outflow in the tumor.

As in the case of patient KIA (Figure 3), intense fluorescence of endogenous protoporphyrin IX (PpIX) was registered in all studied zones (Figure 6) before Ce6 administration in patient PCM, with a fluorescence maximum of about 705 nm [59]. The accumulation of endogenous PpIX in tumor cells reflects disturbances in heme synthesis that are characteristic of many malignant neoplasms and can serve as an indicator of mitochondrial dysfunction [60].

**Figure 7:**
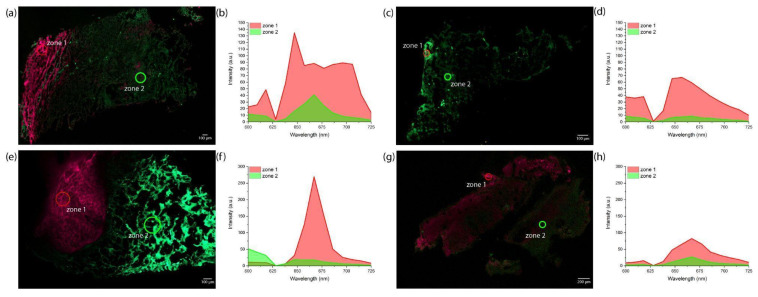
The results of confocal microscopy of the patient’s PCM tumor biopsy specimens after the intra-arterial administration of Ce6: (**a**) fluorescence image of the tumor central zone before PDT; (**b**) fluorescence spectra of the tumor center before PDT; (**c**) fluorescence image of the tumor central zone after PDT; (**d**) fluorescence spectra of the tumor central zone after PDT; (**e**) fluorescence image of the tumor border zone before PDT; (**f**) fluorescence spectra of the tumor periphery before PDT; (**g**) fluorescence image of the tumor border zone after PDT; (**h**) fluorescence spectra of the tumor periphery after PDT. Spectral data are presented as averaged values in the highlighted areas. The green color in the images represents the intensity of autofluorescence in the 500–620 nm range, which is higher than the fluorescence of Ce6 or endogenous PpIX in the 640–725 nm range. The red color in the images represents the intensity of Ce6 fluorescence, which is higher than the autofluorescence.

Confocal scanning microscopy confirmed Ce6 accumulation in the central and border zones of the tumor. The distribution of Ce6 was examined microscopically in tumor biopsies taken from the central (Figure 7a,b) and border (Figure 7e,f) zones approximately 20 min after the start of intra-arterial PS administration.

Intense Ce6 and endogenous PpIX fluorescence was registered in the biopsies of the central tumor zones (Figure 7a,b). The biopsies of the tumor border exhibited the highest Ce6 fluorescence intensity (Figure 7e,f), which corresponds with the results of spectral-fluorescence diagnostics (Figure 6b,c). After superficial and interstitial PDT, a general decrease in Ce6 fluorescence intensity was observed in biopsies taken from the central (Figure 7c,d) and border (Figure 7g,h) zones of the tumor due to photobleaching.

**Figure 8:**
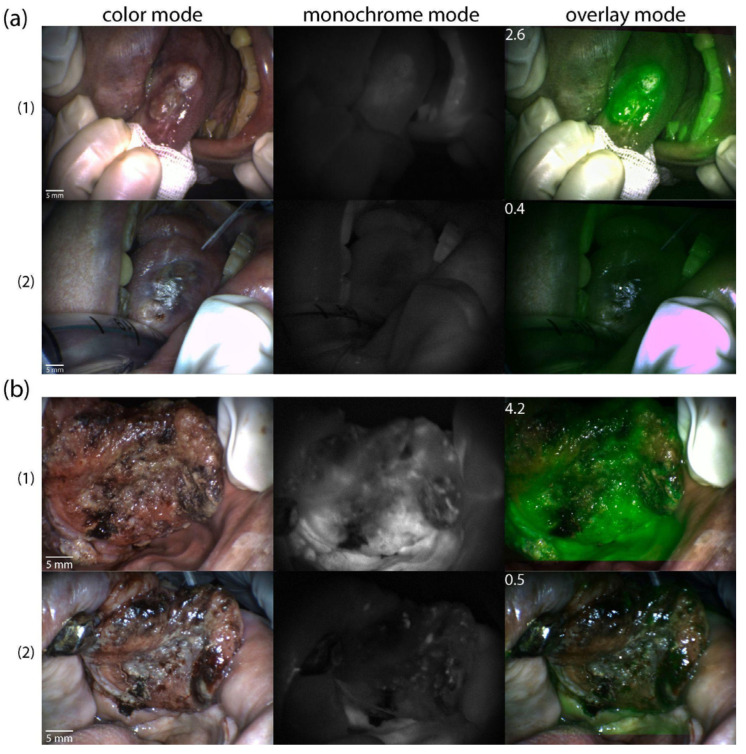
Video fluorescence imaging of tumors after intra-arterial Ce6 administration (**1**) before PDT and (**2**) immediately after PDT in three imaging modes (color, monochrome, and overlay): (**a**) patient KIA; (**b**) patient PCM. Images obtained during the first session of the combination treatment. The contrast indices in the area of the highlighted marker are indicated in the upper left corner of the overlay mode images.

The distribution of Ce6 in tumor tissues before and after PDT was assessed in both patients using video fluorescence imaging. Images were recorded in three modes (color, monochrome, and overlay) and displayed with a contrast index (Figure 8).

Video fluorescence imaging showed high selectivity of Ce6 accumulation in the tumor in both patients. The tumor contrast index was 2.6 ± 0.2 relative units (rel. un.) before PDT in patient KIA (Figure 8(a1)), and it was 4.2 ± 0.3 rel.un. in patient PCM. (Figure 8(b1)). After PDT, a general decrease in Ce6 fluorescence intensity and contrast indices was observed (0.4 ± 0.2 and 0.5 ± 0.3 rel.un., respectively (Figure 8(a2,b2)).

**Figure 9:**
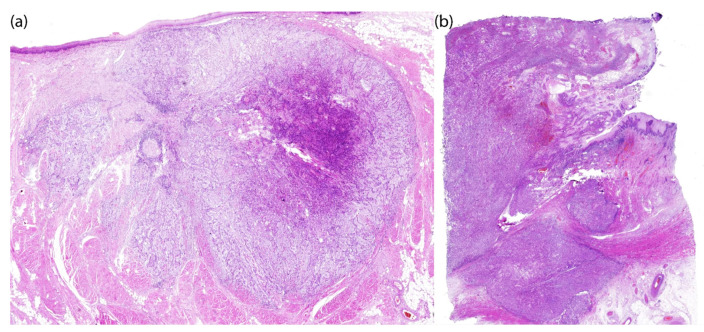
Tumor histology results after combined treatment: (**a**) patient KIA; (**b**) patient PCM. Hematoxylin and eosin staining, ×100 magnification.

The combined use of Ce6-mediated PDT and cisplatin-based chemotherapy produced a significant therapeutic effect in both patients, evidenced by a notable pathomorphological response of the tumor. Histological examination of patient KIA revealed therapeutic pathomorphosis of grades II and III, with dystrophic changes in tumor cells in the invasive growth zone. This corresponded to an almost complete morphological response to combined treatment (Figure 9a).

**Figure 10:**
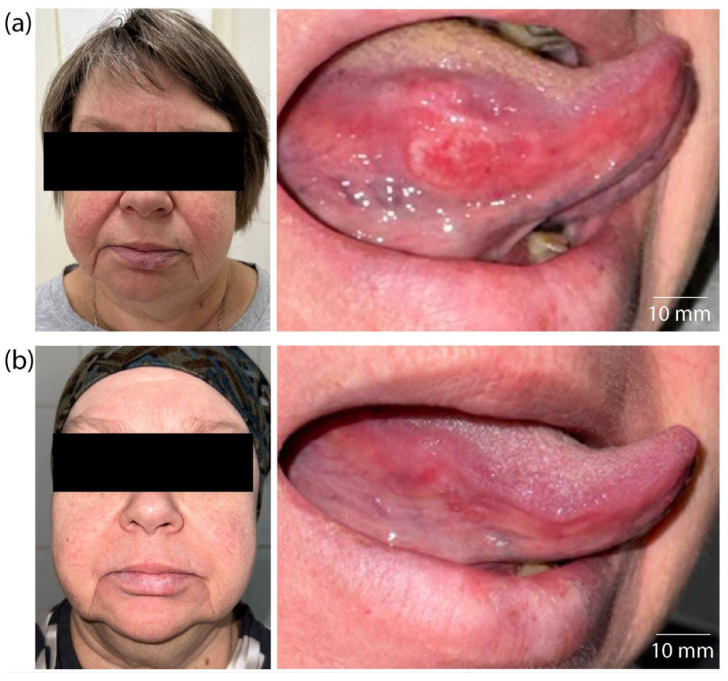
Images of patient KIA: (**a**) before treatment; (**b**) after three months of the combination treatment.

In addition to the histological criteria used to evaluate therapeutic effectiveness, positive changes in patient KIA were observed during a visual assessment of the lesion on the lateral surface of the tongue (Figure 10).

Before treatment, a large infiltrative–ulcerative lesion of the tongue’s mucous membrane with clear signs of inflammation and hyperemia was observed (Figure 10a). After treatment, a significant decrease in the tumor’s size, an absence of inflammatory signs, and a restoration of the mucous membrane’s relief and color were noted (Figure 10b). The absence of visual signs of tumor growth confirms a clinical response and correlates with histopathological data.

**Figure 11:**
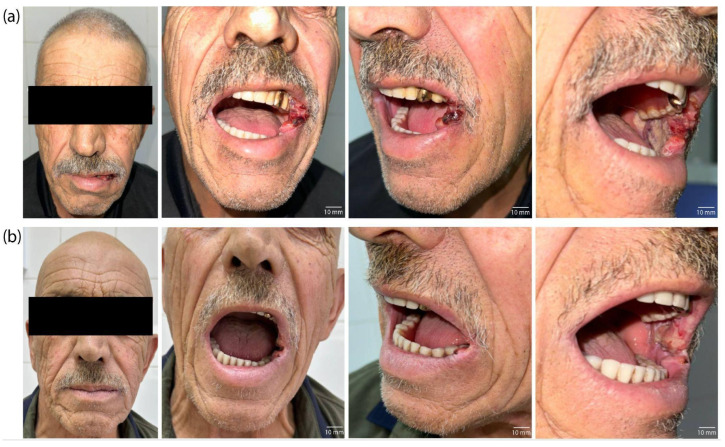
Images of patient PCM: (**a**) before treatment; (**b**) after one month of combination treatment.

Histological examination of the patient’s PCM tissue revealed mild to moderate pathomorphism, with necrosis of more than half of the tumor tissue. Therapeutic pathomorphism of grades I and II was morphologically noted, indicating a partial therapeutic effect. This is evidenced by areas of tumor necrosis and tissue destruction (Figure 9b). Thus, the combined use of PDT and chemotherapy resulted in significant morphological damage to the tumor, as well as an absence of tumor remnants at the resection margins. This absence is a positive prognostic factor. The clinical efficacy of the combined therapy in patient PCM was confirmed by visual examination data (Figure 11).

Before treatment, patient PCM had an extensive tumor lesion with infiltrative–ulcerative changes in the buccal mucosa area, extending to the corner of the mouth and the surrounding soft tissues (Figure 11a). Signs of inflammation, swelling, hyperemia, and tissue destruction were extensive. After treatment, significant improvements were observed: epithelialization of the lesion, absence of active tumor growth, and restoration of mucosal and skin integrity (Figure 11b). The decrease in the volume of the pathological lesion and the absence of signs of infiltration into the surrounding tissues correlate with histological examination data, indicating high-grade (Grade III) therapeutic pathomorphosis. Thus, the visual dynamics confirm tumor regression with combination therapy and illustrate a clinically significant response without significant cosmetic or functional defects.

The obtained results demonstrate the need for larger clinical trials to evaluate the method’s effectiveness, optimize dosages, and expand its applications. Long-term observations will enable us to evaluate the approach’s impact on relapse rates and overall survival.

## 3. Discussion

The results of this preliminary study demonstrate the high clinical efficacy and potential of combining Ce6-mediated PDT and cisplatin-based chemotherapy with the local intra-arterial administration of a photosensitizer and a chemotherapeutic agent for patients with HNSCC. Unlike traditional intravenous administration, which provides systemic distribution of the substances, intra-arterial access creates an increased local concentration of the agents directly in the tumor area while reducing systemic toxicity. Selective catheterization of the tumor-feeding artery ensured targeted administration of both the PS and the chemotherapy agent, making it possible to significantly reduce their dosages: 0.5 mg/kg for Ce6 and 50 mg/m^2^ for cisplatin (versus traditionally used doses of 1–2.5 mg/kg [61,62] and 100 mg/m^2^ [63], respectively).

Intra-arterial infusion provides increased local delivery to the feeding tumor and reduces systemic exposure due to the “first pass” effect, which makes it possible to obtain therapeutic tissue concentrations with a lower total dose [64,65]. Unlike intravenous administration, which leads to systemic distribution and dilution, intra-arterial administration delivers the PS and the chemotherapy agent directly into the tumor-feeding artery, creating a high local concentration in the tumor while minimizing systemic exposure.

This approach is particularly effective when combined with the enhanced permeability and retention (EPR) phenomenon, a pathophysiological phenomenon characteristic of solid tumors that involves selective passive accumulation of macromolecules such as proteins, liposomes, micelles, and other soluble particles larger than 40 kDa in the tumor interstitium and their long-term retention in tissues. This occurs as a functional result of abnormalities including increased vascular permeability, decreased lymphatic drainage, increased ECM stiffness, and high interstitial fluid pressure in tumors [66]. Intra-arterial chemotherapy allows for very high initial drug concentrations in tumor vessels and a decrease in the concentration of the PS and chemotherapy agent entering the systemic circulation. The EPR effect works synergistically with this approach, significantly enhancing its benefits.

The combination strategy itself allows for dose reduction in both components while maintaining or enhancing efficacy, as the mechanisms of action are synergistic and not merely additive [29,67,68].

Using reduced doses of Ce6 and cisplatin in combination with local intra-arterial administration resulted in the therapy being well tolerated. No severe side effects were reported by any of the patients, which is especially important for therapies in the head and neck area. This local administration method demonstrated the high efficiency of Ce6 accumulation in tumor tissue, as confirmed by spectral and video fluorescence visualization and confocal microscopy.

HNSCC has gained notoriety due to its significant ECM stiffness and high stromal infiltration. In particular, increased marginal stiffness has been widely documented in oral squamous cell carcinomas (OSCCs) [69]. The HNSCC microenvironment is collagen-rich, and collagen promotes HNSCC cell proliferation and migration while attenuating the apoptotic response to cisplatin [70]. Discoidin domain receptor 1 (DDR1), a collagen receptor tyrosine kinase-activated protein, is overexpressed in HNSCC tissues and correlates with chemoresistance [71]. Ce6-mediated PDT induces endoplasmic reticulum stress, leading to apoptosis and the release of immunogenic CRT, HSP90, HMGB1, and MHC-I [72]. PDT affects not only tumor cells but also destroys stromal elements by mediating the death of tumor-associated fibroblasts. The resulting cell death, as well as direct DNA damage, causes a cessation of expression and deposition of tumor cell stromal products. These products include ECM molecules such as collagen, hyaluronic acid, laminin, and fibronectin. Consequently, associated desmoplastic and pro-tumorigenic signaling pathways are restricted, reducing tumor cell proliferation, migration, and survival while improving the response to chemotherapy [73].

Thus, PDT, which affects not only tumor cells but also stromal components, mediates a cascade of changes in the TME. Depending on their concentration, Ce6 molecules can inhibit the transport activity of the ABCG2 transport protein [74]. ABCG2 actively removes various PSs from tumor cells, including Ce6 [75], which can reduce the effectiveness of PDT. Inhibiting the ABCB1 or ABCG2 transport proteins can abolish chemoresistance and increase the death of chemoresistant cancer stem cells [76]. In this study, the local intra-arterial administration of PSs enables the temporary creation of a high Ce6 concentration in the TME. This can help overcome the active transport barrier, thereby increasing the intracellular accumulation of the PS and the chemotherapy agent.

In this study, no direct experiments were performed to verify the proposed mechanisms of ECM alteration or ABCG2 modulation. Therefore, conclusions about the contribution of ECM density reduction and ABC transporter inhibition are based on indirect results and literature data rather than on our own morphological or molecular measurements. In particular, it was previously demonstrated that ABCG2 reduces Ce6 accumulation and reduces the efficiency of PDT, and that the selective inhibitor KO143 is able to restore Ce6 accumulation and enhance the effect of PDT [74]. Direct confirmation of the proposed mechanisms will be the subject of further studies and will allow us to quantify the contribution of each mechanism to the observed synergism.

Combining chemotherapy with PDT enhances the antitumor effect due to synergistic action. This reduces the doses of chemotherapy agents and PSs. PDT increases tumor sensitivity and enables it to overcome resistance [77], but its use is limited by the depth of light penetration. Chemotherapy, in turn, has a broad effect on the entire tumor volume [78], but it can cause serious side effects and lose effectiveness as the tumor forms resistance [79]. Combining these methods compensates for these shortcomings, making therapy more effective and safer.

## 4. Materials and Methods

### 4.1. Chemotherapy

Patient KIA received three courses of combination therapy involving intra-arterial cisplatin chemotherapy, while patient PCM received one course. Both patients also received systemic docetaxel. During the intra-arterial chemotherapy (superselective chemoinfusion) procedure, the patients received 50 mg/m^2^ of 0.5% cisplatin. The cisplatin was infused within approximately 60 min through an arterial catheter via an infusion pump at a rate of 5 mL/min. Then, docetaxel was administered intravenously over one hour at a dosage of 75 mg/m^2^ in 500 mL of physiological sodium chloride solution. The next course began on day 21. Treatment was carried out against the background of premedication: Dexamethasone (8 mg) was administered intramuscularly the day before docetaxel administration at 11:00 p.m. and again on the day of treatment at 5:00 a.m. Antiemetics (Ondansetron 16 mg intravenously) and antihistamines (Suprastin 25 mg intravenously) were also administered. To prevent nephrotoxicity, prehydration with 2.0 L of 0.9% sodium chloride solution was performed with forced diuresis (Mannitol) before cisplatin administration.

### 4.2. Tumor Catheterization

Catheterization was performed through a transfemoral approach with the installation of a 6 F introducer through the right superficial femoral artery. Under fluoroscopic control and using the contrast agent Omnipaque^®^ (GE Healthcare, Chicago, IL, USA) (Iopamidol 300 mg/mL), a JR 5 F catheter was inserted into the left ECA for patient PCM and into the area of the mouth of the facial and lingual arteries of the right ECA for patient KIA, both along the guidewire. Selective catheterization of the tumor-feeding artery was then performed using a 2.4 F microcatheter (Terumo, Tokyo, Japan), followed by angiographic verification of the position. The average fluoroscopy time was 17 min.

### 4.3. Photosensitizer

For fluorescence diagnostics and PDT, we used a commercially available and clinically approved drug: Photoran E6^®^ (DEKO Company Ltd., Moscow, Russia). The PS was administered at a calculated dose of 0.5 mg/kg of body weight, dissolved in 5 mL of a 0.9% sodium chloride solution. It was bolus administered through an intra-arterial catheter. After the administration of the PS, patients followed a light regimen for 24 h, avoiding direct sunlight exposure to their skin and eyes. Starting on the day of administration and continuing for three days, sunscreen cream was applied to exposed skin areas.

### 4.4. Photodynamic Therapy

**Figure 12:**
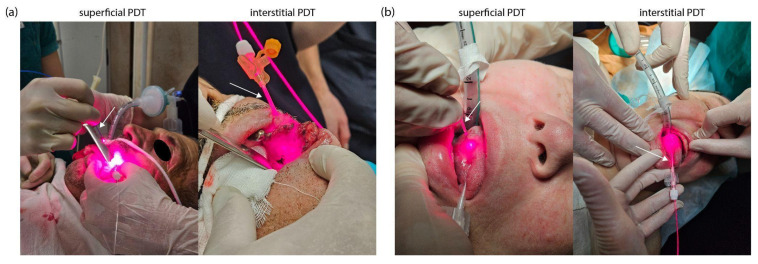
PDT process involving superficial and interstitial laser exposure of the tumor: (**a**) patient PCM; (**b**) patient KIA.

For PDT, a semiconductor laser LFT-02-BIOSPEC (BIOSPEC Ltd., Moscow, Russia) with a wavelength of 663 ± 5 nm and a maximum output power of 2000 mW was used. Before PDT, all patients were given total intravenous anesthesia using propofol at a dose of 2 mg/kg and fentanyl at a dose of 2 mcg/kg. PDT was performed in two stages: first, superficial laser irradiation of the tumor was performed, then interstitial PDT was performed with the location of cylindrical diffuser directly in the tumor tissue (Figure 12).

Laser radiation delivery during superficial PDT was performed using a quartz optical fiber (BIOSPEC Ltd., Moscow, Russia) with a core diameter of 600 μm and a numerical aperture of NA = 0.22. A microlens installed at the distal end of the fiber ensured uniform light distribution, reducing unevenness to less than 10%.

For interstitial PDT, we used a flexible polymer optical fiber (BIOSPEC Ltd., Moscow, Russia) with a 400 μm diameter and a 20 mm long cylindrical diffuser. Laser irradiation was performed in polyposition mode. The PDT session was completed when the fluorescence intensity of Ce6 decreased by 70–80% from the initial level. Photobleaching of Ce6 was assessed by spectral-fluorescence diagnostics based on the decrease in the integral fluorescence intensity in the spectral range of 650–690 nm and video fluorescence imaging based on the change in the fluorescence contrast indices. The light fluence rates and total energy doses were precisely set and controlled by the laser system (LFT-02-BIOSPEC). The parameters were for superficial PDT: a power density of 645 mW/cm^2^ was delivered to the surface, with a total energy dose of 130–150 J/cm^2^; for interstitial PDT: the cylindrical diffuser emitted a power density of 356 mW/cm (linear power density along the diffuser length), with a total energy dose of 80–100 J/cm^2^.

### 4.5. Spectral-Fluorescence Diagnostics

Spectral-fluorescence diagnostics were performed using the LESA-01-BIOSPEC spectroscopic system (BIOSPEC Ltd., Moscow, Russia). Ce6 fluorescence was excited using a helium–neon (He-Ne) laser (BIOSPEC Ltd., Moscow, Russia) with a wavelength of 632.8 nm and an output power of 5–6 mW. Spectra were recorded using six receiving optical fibers (BIOSPEC Ltd., Moscow, Russia) that were uniformly distributed around the central fiber, which was designed to deliver the exciting laser radiation. All fibers had a diameter of 125 μm and a numerical aperture of NA = 0.22. The spectral signal was recorded in the 600–800 nm range, including the backscattering region of laser radiation and Ce6 fluorescence. The spectra were normalized to the maximum value of the intensity of backscattered laser radiation. Spectral-fluorescence diagnostics were performed in three anatomical and functional zones: normal tissue, the central part of the tumor, and the tumor border (5 ± 1 mm from the visible edge).

The exposure time for recording spectra ranged from 20 to 100 ms, depending on the signal intensity. At least five spectra were recorded at each time point and averaged to improve measurement accuracy. The integral fluorescence intensity was calculated for each zone as the area under the spectral curve in the range of 650–690 nm.

### 4.6. Video Fluorescence Imaging

The VENERA-red dual-channel fluorescence video system (BIOSPEC Ltd., Moscow, Russia) was used to detect tumor boundaries and quantitatively assess the distribution of Ce6 in tissues. The system includes a white light source, a semiconductor laser with a wavelength of 635 nm, a Y-shaped light-guide cable, and an endoscopic recording unit with highly sensitive black-and-white and color CCD cameras and an endoscope.

Fluorescence visualization can be performed in three modes: color, monochrome, and overlay. In overlay mode, software synchronizes the operation of the two cameras and superimposes a monochrome image of fluorescent areas (λ > 650 nm) onto a color image in real time. Pathological tissue visualization was performed by displaying fluorescent foci in green, which allowed for the differentiation of tumor and healthy tissues and the calculation of the fluorescence contrast coefficient. The method for calculating the fluorescence contrast coefficient is presented in the study [80], in which the contrast coefficient is preliminarily defined by the fluorescence intensity of normal tissue (1 rel.un.). This allows identification of zones with increased PS concentration, with a contrast coefficient > 1 rel.un.

### 4.7. Confocal Microscopy

Microscopic examination of Ce6 distribution in tumor tissues was performed using confocal microscopy (Carl Zeiss Microscopy GmbH, Jena, Germany) before and after PDT. No radical surgical removal of the tumor was performed during biopsy; targeted biopsy was used to obtain the material. Each biopsy was no larger than 3 × 3 × 3 mm. After collection, the sample was placed in an isotonic NaCl solution (0.9%) and examined for 24 h.

Prior to microscopy, the biopsies were embedded in Neg-50 freezing medium (Richard-Allan Scientific, Kalamazoo, MI, USA) and frozen until solid. Twenty-micrometer-thick cryostat sections were made using a Microm HM 540 apparatus (Thermo Fisher Scientific Inc., Waltham, MA, USA). The sections were applied to microscope slides, embedded in glycerol under a cover glass, and examined without additional staining. Spectral analysis of fluorescence was performed using an LSM-710 NLO laser scanning confocal microscope (Carl Zeiss Microscopy GmbH, Jena, Germany). Tissue autofluorescence was excited by a 488 nm laser, and Ce6 fluorescence was excited by a 633 nm laser. Plan-Apochromat 20×/0.8 (Carl Zeiss Microscopy GmbH, Jena, Germany) and Plan-Apochromat 63×/1.4 (Carl Zeiss Microscopy GmbH, Jena, Germany) objectives with oil immersion were used for visualization.

### 4.8. Histology

A histological examination of the degree of therapeutic pathomorphosis after neoadjuvant therapy was carried out according to a modified classification with a quantitative assessment of tumor cellularity [81]. Grade I: weak therapeutic pathomorphosis; total tumor cellularity greater than 70%; no reduction or only minor loss of tumor cells (partial response, PR). Grade II: moderate therapeutic pathomorphosis; cellularity reduced from 10 to 70% of total tumor cellularity; appearance of foci of necrosis and dystrophic changes in tumor cells. Grade III: indicating high-grade (Grade III) therapeutic pathomorphosis with less than 10% total tumor cellularity, extensive necrosis, and pronounced dystrophic changes in tumor cells with a few viable tumor cells remaining. There are also foci of carcinoma in situ. Grade IV: pronounced, complete therapeutic pathomorphosis with an absence of tumor elements and macrophage infiltration in the fibrovascular stroma (complete response, CR).

### 4.9. Statistical Analysis

The results of differences in fluorescence intensity in different zones are presented in the format of “mean ± standard deviation” (mean ± SD). The significance of differences in fluorescence intensity of different study zones was tested using the paired Student’s *t*-test. The critical level of significance for confirming the reliability of differences was set at *p* < 0.05.

## 5. Conclusions

The results of this pilot study demonstrate the potential of combined use of PDT and chemotherapy with local intra-arterial administration of Ce6 and cisplatin for the treatment of HNSCC. Selective intra-arterial administration ensured the targeted accumulation of the PS and chemotherapy agent in tumor tissue. Simultaneously, reduced doses were used (0.5 mg/kg of Ce6 and 50 mg/m^2^ of cisplatin) compared to standard systemic treatment regimens without compromising the therapeutic effect.

Both patients showed a pronounced antitumor response after therapy. Patients demonstrated significant tumor volume reduction and concomitant tissue regeneration following combination therapy. The combination of Ce6-mediated PDT and cisplatin chemotherapy demonstrated significant effects on tumor ECM remodeling. This clinically significant response enabled the patients to transition to an operable condition without significant functional or cosmetic defects, providing the possibility of further radical surgical treatment that preserves anatomical integrity and physiological function.

The combined use of Ce6-mediated PDT and cisplatin-based chemotherapy, with the local intra-arterial administration of the PS and chemotherapy agent, showed good tolerability without severe side effects. This innovative approach is an effective method of treating tumors and opens up new possibilities for improving the treatment of oncological diseases.

## 6. Patients

The study was conducted from March to May 2025 at the Department of Oncology, Radiotherapy and Reconstructive Surgery of the I.M. Sechenov First Moscow State Medical University. Two patients with HNSCC were included in the study. The patients underwent neoadjuvant therapy to reduce the size of the primary tumor, allowing for R0 surgery and organ-preserving treatment (Table 2).

Patient PCM was admitted with a diagnosis of C06.0, malignant neoplasm of the buccal mucosa. Based on the results of the pre-therapy histological examination, the diagnosis was invasive squamous cell keratinizing carcinoma with a differentiation degree of G2. The tumor immunophenotype was p16 negative, p53 positive (with expression in 60% of tumor cell nuclei), Ki-67 70%, and PD-L1 (DAKO 22C3) CPS 20 TC. The clinical stage of the disease was T3N0M0. A cytological examination of a left cervical lymph node puncture revealed no signs of a tumor lesion.

Patient KIA was admitted with a diagnosis of C02.1, malignant neoplasm of the lateral surface of the tongue. According to the histological conclusion, keratinizing squamous cell carcinoma of the tongue (stage T2N0M0) was detected before the start of treatment. A cytological examination of the cervical lymph node tissue preparation on the right did not reveal signs of tumor damage; rather, hyperplasia of lymphoid elements was diagnosed.

Both patients underwent a combination treatment with Ce6-mediated PDT and cisplatin chemotherapy, including intra-arterial administration of a PS and chemotherapy agent. All patients signed informed consent to participate in the study.

Since our study is the first to examine the combined use of Ce6-mediated PDT and cisplatin-based chemotherapy with intra-arterial administration of a PS and chemotherapeutic agent, our primary objective is to evaluate safety, technical feasibility, and basic pharmacodynamic parameters. Early “pilot” trials often include a small number of patients to minimize risks and resources when launching an innovative protocol [82]. Formal power calculations are not required in pilot studies; the sample size is chosen based on the goal of practicing the procedure, assessing tolerability, identifying possible technical problems, and obtaining initial data for a larger subsequent study [83].

## Figures and Tables

**Table 1:** Clinical studies of the combined use of PDT and chemotherapy.

Study	PS	Chemotherapeutic Agent	Administration Method	Cancer Type	Key Findings
Wentrup R. et al., 2016 [40]	Photofrin II^®^	Cisplatin/Oxaliplatin/Gemcitabine	Intravenous (PS) + Systemic (Chemo)	Hilar NCC	Combined therapy improved survival vs. PDT alone.
Gonzalez-Carmona M. A. et al., 2019 [54]	Photosan^®^/Photofrin^®^/Foscan^®^ (Temoporfin, mTHPC)	Gemcitabine/Cisplatin	Intravenous (PS) + Systemic (Chemo)	Advanced EHCC	Combination PDT and chemotherapy was well tolerated and resulted in significantly longer survival than chemotherapy alone.
Zhang N. Z. et al., 2007 [55]	Photocarcinorin (PSD-007)	5-fluorouracil	Intravenous (PS) + Local Intra-Arterial (Chemo)	Advanced Esophagocardiac Carcinoma	Therapeutic effect of the PDT can be further improved when combined with local chemotherapy.
Hong M. J. et al., 2013 [56]	Photofrin^®^	Gemcitabine/Cisplatin	Intravenous (PS) + Systemic (Chemo)	Advanced HCC	PDT with chemotherapy results in longer survival than PDT alone.
**Current study**	**Ce6** **(Photoran E6^®^)**	**Cisplatin**	**Local Intra-Arterial (PS) +** **Local Intra-Arterial (Chemo)**	**Advanced HNSCC**	**Targeted delivery of both agents (PS and Chemo), reduced doses, and showed high efficacy and safety.**

Abbreviations: PS—photosensitizer; PDT—photodynamic therapy; Chemo—chemotherapeutic agent; NCC—nonresectable cholangiocarcinoma; EHCC—extrahepatic cholangiocarcinoma; HCC—hilar cholangiocarcinoma; Ce6—chlorin e6; HNSCC—head and neck squamous cell carcinoma.

**Table 2:** Main patient parameters.

Parameter	Patient PCM	Patient KIA
Gender	male	female
Age	64 years	70 years
Weight	63 kg	103 kg
Body Mass Index	23.7	39.2

## Data Availability

The data presented in this study are available on request from the corresponding author.

## Abbreviations

HNSCC	Head and neck squamous cell carcinoma
PDT	Photodynamic therapy
Ce6	Chlorin e6
PS	Photosensitizer
WHO	World Health Organization
ECM	Extracellular matrix
TME	Tumor microenvironment
ROS	Reactive oxygen species
Pt(II)	Platinum(II)
CDDP	Cis-diamminedichloridoplatinum(II)
CBDCA	cis-diammine-1,1-cyclobutanedicarboxylatoplatinum(II)
1-OHP	trans-R,R-cyclohexane-1,2-diamineoxalateplatinum(II)
DNA	Deoxyribonucleic acid
NCC	Nonresectable cholangiocarcinoma
EHCC	Extrahepatic cholangiocarcinoma
HCC	Hilar cholangiocarcinoma
9-HPbD	9-hydroxypheophorbide α
mTHPC	Meta-tetra(hydroxyphenyl)chlorin
ZnPc	Zinc phthalocyanine
DOX	Doxorubicin
CT	Computed tomography
ECA	External carotid artery
PpIX	Protoporphyrin IX
EPR	Enhanced permeability and retention
OSCCs	Oral squamous cell carcinomas
DDR1	Discoidin domain receptor 1
PR	Partial response
CR	Complete response
